# High Prevalence of Antibodies against the Bacterium *Treponema pallidum* in Senegalese Guinea Baboons (*Papio papio*)

**DOI:** 10.1371/journal.pone.0143100

**Published:** 2015-11-20

**Authors:** Sascha Knauf, Ulrike Barnett, Peter Maciej, Matthias Klapproth, Ibrahima Ndao, Sieghard Frischmann, Julia Fischer, Dietmar Zinner, Hsi Liu

**Affiliations:** 1 Work Group Neglected Tropical Diseases, Pathology Unit, German Primate Center, Leibniz-Institute for Primate Research, Göttingen, Germany; 2 Cognitive Ethology Laboratory, German Primate Center, Leibniz-Institute for Primate Research, Göttingen, Germany; 3 Direction du Parc National Niokolo Koba, Tambacounda, Senegal; 4 Mast Diagnostica GmbH, Reinfeld, Germany; 5 National Center for HIV/AIDS, Viral Hepatitis, STD, and TB Prevention, Centers for Diseases Control and Prevention, Atlanta, Georgia, United States of America; Jamia Millia Islamia, INDIA

## Abstract

The bacterium *Treponema pallidum* is known to cause syphilis (ssp. *pallidum*), yaws (ssp. *pertenue*), and endemic syphilis (ssp. *endemicum*) in humans. Nonhuman primates have also been reported to be infected with the bacterium with equally versatile clinical manifestations, from severe skin ulcerations to asymptomatic. At present all simian strains are closely related to human yaws-causing strains, an important consideration for yaws eradication. We tested clinically healthy Guinea baboons (*Papio papio*) at Parc National Niokolo Koba in south eastern Senegal for the presence of anti-*T*. *pallidum* antibodies. Since T. pallidum infection in this species was identified 50 years ago, and there has been no attempt to treat non-human primates for infection, it was hypothesized that a large number of West African baboons are still infected with simian strains of the yaws-bacterium. All animals were without clinical signs of treponematoses, but 18 of 20 (90%) baboons tested positive for antibodies against *T*. *pallidum* based on treponemal tests. Yet, Guinea baboons seem to develop no clinical symptoms, though it must be assumed that infection is chronic or comparable to the latent stage in human yaws infection. The non-active character is supported by the low anti-*T*. *pallidum* serum titers in Guinea baboons (median = 1:2,560) versus serum titers that are found in genital-ulcerated olive baboons with active infection in Tanzania (range of medians among the groups of initial, moderate, and severe infected animals = 1:15,360 to 1:2.097e+7). Our findings provide evidence for simian infection with *T*. *pallidum* in wild Senegalese baboons. Potentially, Guinea baboons in West Africa serve as a natural reservoir for human infection, as the West African simian strain has been shown to cause sustainable yaws infection when inoculated into humans. The present study pinpoints an area where further research is needed to support the currently on-going second WHO led yaws eradication campaign with its goal to eradicate yaws by 2020.

## Introduction

In the mid-1960s, serological surveys demonstrated that the bacterium *Treponema pallidum* infected large numbers of nonhuman primates in Guinea Bissau, Senegal, and Cameroon [1–4]. While the bacterium, which belongs to the order of Spirochaetales, is known to cause syphilis (ssp. *pallidum*), yaws (ssp. *pertenue*, TPE), and endemic syphilis (ssp. *endemicum*) in humans, simian isolates have been reported to cause equally versatile clinical manifestations (reviewed in [5]). At present, all simian isolates are genetically most closely related to human yaws-causing strains [6, 7]. Currently the Fribourg-Blanc simian strain, which was isolated from a baboon in West Africa [4], is the most profoundly characterized simian isolate. It has been whole genome sequenced and due to its similar genetic characteristics, matching those of other TPE strains, it has been proposed to be renamed as *T*. *pallidum* ssp. *pertenue* strain Fribourg-Blanc [6]. It is furthermore the only simian strain that has been shown to infect humans when inoculated into skin [8], though it should be noted that the indicated study must be considered as ethically questionable. Nevertheless, the findings suggest that simian strains may successfully cross species barriers, an important observation for the ongoing second WHO led yaws eradication campaign [9]. In the 1960’s, the isolated West African simian strain was described to cause mild skin lesions in some baboons that included small keratotic lesions and ulcers on the muzzle, eyelids, and armpits. However, most serologically positive animals were free of any clinical symptoms [2].

We tested clinically healthy Guinea baboons (*Papio papio*) at Parc National Niokolo Koba (PNNK) in south eastern Senegal for the presence of anti-*T*. *pallidum* antibodies. It was hypothesized that infection is present even five decades after its first description in West Africa, especially because the baboons had no history of treatment against the spirochete in this area.

## Material and Methods

### Ethical statement

All animal work was conducted according to relevant national and international guidelines. Baboon serum samples were taken with permission of the National Parks Direction and the Ministry of Environment and Sustainable Development of Senegal (Attestation 0383/24/03/2009, 0373/10/03/2011, and 1089/02/09/2013). In addition, the Animal Welfare and Ethics Committee of the German Primate Center approved the entire study. ‘Good Veterinary Practice’ rules were applied to all procedures whenever animals were handled, e.g. during blood sampling. A veterinarian closely monitored anaesthetized animals until they were fully recovered and able to make their way back to their group.

### Study site and animals

PNNK is located in the south eastern part of Senegal and borders Guinea to the south. While poaching threatens several species of native wildlife in the park and has reduced their numbers, the park’s population of Guinea baboons is stable and on the rise [10]. The study area lies next to the ‘Centre de Recherche de Primatologie’ (CRP) at Simenti (GPS N13.026111, W13.294722), which is located next to the Gambia River. The field site is operated by the German Primate Center, and its main focus is on the behavioral ecology, social system, and cognition of Guinea baboons in their natural environment. The study population consists of ~300 baboons of which ~150 are habituated to the close presence of human observers [11]. The home range encompasses about 25 km^2^ [11]. Since 2007, behavioral research has been ongoing with students and park rangers performing daily focal observations, following the habituated baboons from 6:00 AM– 12:00 PM and 4:00 PM– 7:00 PM. To track the whereabouts of the different study groups, a number of adult female and male baboons were collared e.g., with VHF radio transmitters collar devices (M2320, 130 g, ATS, Isanti, MN, USA). In order to collar, remove or exchange transmitters, 20 animals, 4 females and 16 males, underwent routine anesthesia, which allowed access for blood sampling in April to May 2013 (n = 12) and November to December 2014 (n = 9; Table 1, one baboon was captured in 2013 and 2014 and is counted in total as a single individual).

**Table 1:** Spatial-, demography- and life-time data of animals that were sampled for blood. GPS data indicate the sampling site. All baboons were clinically healthy. The baboon ID ends with the date of sampling. n/m = not measured.

Case	Baboon ID	Sampling Site (GPS Data, Decimal Degrees, N and W)	Body Weight (kg)	Sex
1	3-OSM-25.04.13*	13.03314, -13.28127	20.0	Male
2	4-MST-25.04.13	13.02652, -13.29628	23.0	Male
3	5-MSA-26.04.13	13.02549, -13.29602	22.0	Male
4	6-AMT-28.04.13	13.02959, -13.27823	12.0	Female
5	7-HOK-28.04.13	13.02892, -13.27821	20.0	Male
6	8-SNE-29.04.13	13.01826, -13.28522	21.5	Male
7	9-NDO-30.04.13	13.00957, -13.27582	20.0	Male
8	10-JLA-01.05.13	13.02788, -13.28452	14.0	Female
9	11-BNT-02.05.13	13.03016, -13.28543	n/m	Female
10	15-JHN-09.05.13	13.02344, -13.28742	21.0	Male
11	16-MRM-10.05.13	13.03697, -13.27967	10.0	Female
12	18-FRD-12.05.13	13.02019, -13.28528	22.0	Male
13	1-RBT-23.11.14	13.03775, -13.31649	20.0	Male
14	2-ANT-24.11.14	13.02555, -13.29417	20.5	Male
15	3-FDL-25.11.14	13.02559, -13.29420	20.0	Male
16	4-NDR-26.11.14	13.02559, -13.29420	20.5	Male
17	5-DRK-30.11.14	13.02555, -1329421	19.5	Male
18	6-OSM-01.12.14*	13.02559, -13.29420	19.0	Male
19	9-JKY-07.12.14	13.02558, -13.29417	22.5	Male
20	10-BAA-11.12.14	13.02788, -13.28452	20.0	Male
21	11-FDR-12.12.14	13.01089, -13.26952	21.0	Male

*The same animal sampled in 2013 and 2014

Continuous health data are available from all baboons of the habituated study group. Signs of discomfort (e.g., fatigue or reduced grooming behavior), lesions caused by trauma, or chronic skin alterations or rash were recorded and reported on a regular basis.

### Anesthesia and sampling

Baboons were either short-term immobilized while they were ranging in their group, or when trapped in a custom-made baboon trap. Chemical immobilization was achieved using a mixture of 5.5 mg ketamine/kg body weight (bm) (Ketavet, Pfizer, Berlin, Germany), 1.1 mg xylazine/kg bw (Rompun, Bayer, Leverkusen, Germany), and 0.01 mg atropine/kg bm (Atropium sulfuricum, Eifelfango, Bad Neuenahr-Ahrweiler, Germany). Injection was done by intramuscular remote distance injection using blowpipe or cold-gas immobilization rifle.

Body weight was measured using a spring scale and animals were monitored for vital parameters, such as breath and heart frequency as well as internal body temperature, followed by a routine health check with special focus on skin lesions such as chronic skin ulcers, rash, or bone deformation.

Blood sampling was performed away from the other baboons and in the shade. After proper disinfection of the skin the femoral vein was punctured using a closed blood collection system with a 20G needle (S-Monovette-Kanüle 20Gx1/1.5, #85.1160) to protect the researcher from direct contact with blood. Briefly, a total of 27 ml whole blood was collected with one Lithium-Heparine (S-Monovette 9 ml LH, #02.1065.001), one EDTA (S-Monovette 9 ml K3E, #02.1066.001), and one serum collection tube (S-Monovette 9 ml Z, #02.1063). After removal of the needle, firm pressure was applied to the puncture side to prevent formation of hematoma. Blood samples were kept cool utilizing a recoolx-bag (Recoolx Sievers, Bramsche, Germany) until proper storage facilities were reached after return to the field camp. Samples were kept in upright position until sedimentation was achieved.

Sample processing occurred in the late evening of the sampling day. Plasma and serum samples were divided into 1.0-ml aliquots and transferred into 1.5-ml Protein LoBind tubes (Eppendorf, Hamburg, Germany). All steps were performed using sterile syringes and needles. Plasma and serum aliquots were stored at approximately -10°C using a gas-operated freezer (Dometic RC 2200 50mbar, Waeco, Emsdetten, Germany) until samples were exported to the German Primate Center, where they were stored at -80°C until they were used for the different serological tests.

### Serology

Validation of serological tests and performance characteristics with baboon sera (sensitivity, specificity, positive and negative predictive values) excluding the detection of cardiolipin can be found elsewhere [12]. Due to sample loss and degradation during export, animals sampled in 2014 were only tested in the field with the treponemal test (TT) ESPLINE TP (Fujirebio Diagnostics Inc., Malvern, PA, USA), whereas baboons sampled in 2013 were tested extensively for the presence of anti-*T*. *pallidum* antibodies as it is described below.

### Treponemal tests

Briefly, serum samples were tested for anti-*T*. *pallidum* antibodies in the field using the ESPLINE TP (Fujirebio Diagnostics Inc., Malvern, PA, USA) TT. Four other TTs were used to detect antibodies against *Treponema* in one of our German laboratories (SF): Syphilitop Optima (ALL. DIAG S.A.S., Strasbourg, France), Mastafluor FTA-ABS IgM and IgG (Mast Diagnostica, Reinfeld, Germany), Mastablot TP (Mast Diagnostica, Reinfeld, Germany), and Serodia TP-PA (Fujirebio Diagnostics Inc., Malvern, PA, USA). The latter was used to assess anti-*T*. *pallidum* titres. All TTs were performed as described elsewhere [12].

### Non-treponemal tests

Non-treponemal tests (NTTs) were used to detect antibodies to lipid antigens and were performed at the German Primate Center (SK). While the RPR-100 (Biorad, Marnes, France) and the VDRLCHECK Charbon/RPR (ALL. DIAG S.A.S., Strasbourg, France) were used as described elsewhere [12], we also included a cardiolipin IgG (cut-off value > 10 U) (Mast Diagnostica, Reinfeld, Germany) and IgM assay (cut-off value > 8 U) (Mast Diagnostica, Reinfeld, Germany) to further evaluate the presence of IgG and IgM-class antibodies against cardiolipins. Cardiolipin IgG and IgM assays were run at Mast Diagnostica, Reinfeld, Germany (SF). Purified cardiolipin antigens of bovine heart origin were bound to an enzyme immunoassay (EIA) solid phase. The assay procedure as well as baboon sera dilution were performed according to the kit’s instruction. For the differentiation between anti-IgG and anti-IgM-antibodies, specific conjugates were used to detect the different antibody-antigen immune complexes. The results were obtained by using an EIA plate reader (Sunrise, Tecan, Männedorf, Switzerland; filter setting (450/620 nm)), calculating the serum concentration from a 4-parameter calibration curve, which was performed for the IgG and IgM assay separately.

### Statistics

Statistical analyses were performed using Prism 6.0 (GraphPad Software, La Jolla, CA, USA). Endpoint titers of exponential scale were log_10_ transformed to reduce variance. In case of non-Gaussian distribution and log transformation, zero-titers were converted into 10e-14. Normal distribution was tested using the D’Agostino & Pearson omnibus normality test and the Shapiro-Wilk normality test. Antibody titers from this study were compared to the results of anti-*T*. *pallidum* titers from a different study [12], which were assorted into four different baboon categories: clinically non-affected, initially, moderately, and severely genital-ulcerated [13]. The non-parametric Kruskal-Wallis test was applied to the log^10^-transformed data sets of the antibody titers. Each mean rank was compared to the mean rank of every other group. Dunn’s correction for multiple comparisons (significance without confidence intervals) was applied to the test. In all tests, p ≤ 0.05 was considered statistically significant.

## Results

### Sero-positivity is not associated with clinical signs of infection

No signs of chronic skin ulceration or rash have been reported from baboons in the study area at PNNK since behavioral research started in 2007. Also, animals that were chemically immobilized were thoroughly clinically inspected and showed no abnormalities that could be associated with *T*. *pallidum* infection. The only skin lesions found were due to acute or partly healed skin wounds from trauma, especially in elderly males.

### High sero-prevalence in Guinea baboons at PNNK

Although all animals were without clinical signs of treponematosis, 18 of 20 (90%) examined baboons were tested positive for antibodies against *T*. *pallidum* based on the outcome of the TTs. The only exception was one female of the 12 animals tested in 2013 and one male baboon of the 9 animals tested in 2014. The female was negative for antibodies against *T*. *pallidum* in all serological tests (case 4, Table 2, S1 Table), while the male baboon in 2014 was only tested using the ESPLINE TP.

**Table 2:** Crosstable of the results of seven treponemal tests and four non-treponemal tests that were used to detect anti-*T*. *pallidum* antibodies in the Senegalese baboons sampled in 2013. Case 13–21 (Table 1) are not included.

		Treponemal Tests							Non-Treponemal Tests			
Case	Baboon ID	Espline TP	Syphili-top Optima	Serodia TP-PA (Titer 1:x)	Mastafluor FTA-ABS IgG	Mastafluor FTA-ABS IgM	Masta-blot TP IgG	Masta-blot TP IgM	VDRL-CHECK	RPR-100	Cardiolipin IgG (Co > 10 U)	Cardiolipin IgM (Co > 8 U)
1	3-OSM-25.04.13	+	+	1,280	+	-	+	-	+	+	11.27	8.82
2	4-MST-25.04.13	+	+	1,280	+	-	+	-	+	-	6.71	3.58
3	5-MSA-26.04.13	+	+	2,560	+	-	+	-	+	+	6.48	6.95
4	6-AMT-28.04.13	-	-	0	-	-	-	-	-	-	5.16	4.99
5	7-HOK-28.04.13	+	+	10,240	+	-	+	-	+	+	6.09	7.96
6	8-SNE-29.04.13	+	+	10,240	+	+	+	-	+	+	7.93	9.80
7	9-NDO-30.04.13	+	+	1,280	+	-	+	-	+	-	7.02	10.59
8	10-JLA-01.05.13	+	+	10,240	+	-	+	-	-	-	4.79	3.73
9	11-BNT-02.05.13	+	+	20,480	+	-	+	-	+	+	8.76	4.13
10	15-JHN-09.05.13	+	+	1,280	+	-	+	-	-	-	4.64	4.27
11	16-MRM-10.05.13	+	+	2,560	+	-	+	+	-	-	5.13	1.79
12	18-FRD-12.05.13	+	+	5,120	+	-	+	-	-	-	5.85	2.84

Results of the Mastafluor FTA-ABS IgG were in accordance to the ESPLINE TP findings, while Mastafluor FTA-ABS IgM detected antibodies of the IgM class against *T*. *pallidum* in only a single baboon (8.3%, case 6, Table 2, S1 Table). The same finding was present when serum was tested with the Mastablot TP for IgG and IgM. Again, the female 6-AMT-28.04.13 (case 4, Table 2, S1 Table) was negative for IgG antibodies against the pathogen, whereas all other baboons tested positive. IgM, however, was detected in only a single animal using Mastablot TP IgM. This animal (case 10, Table 2, S1 Table) was different from the one that was identified to have IgM antibodies against *T*. *pallidum* in the Mastafluor FTA-ABS IgM test (case 6, Table 2, S1 Table).

VDRLCHECK and RPR-100 results differ in two cases (2 and 7, Table 2, S1 Table). In both cases the VDRLCHECK result was positive, while the RPR-100 test result was negative. The female that was negative in all TTs was equally negative in both NTTs. Antibodies of the IgM class directed against cardiolipins exceeded the cut-off value in 3 animals (case 1, 6, and 7, Table 2) and thus were considered to be positive. Only one animal had a positive outcome for IgG anti-cardiolipin antibodies against the spirochete (case 1, Table 2, S1 Table).

### Anti-*T*. *pallidum* antibody titers are not as high as in East African baboons

**Fig 1:**
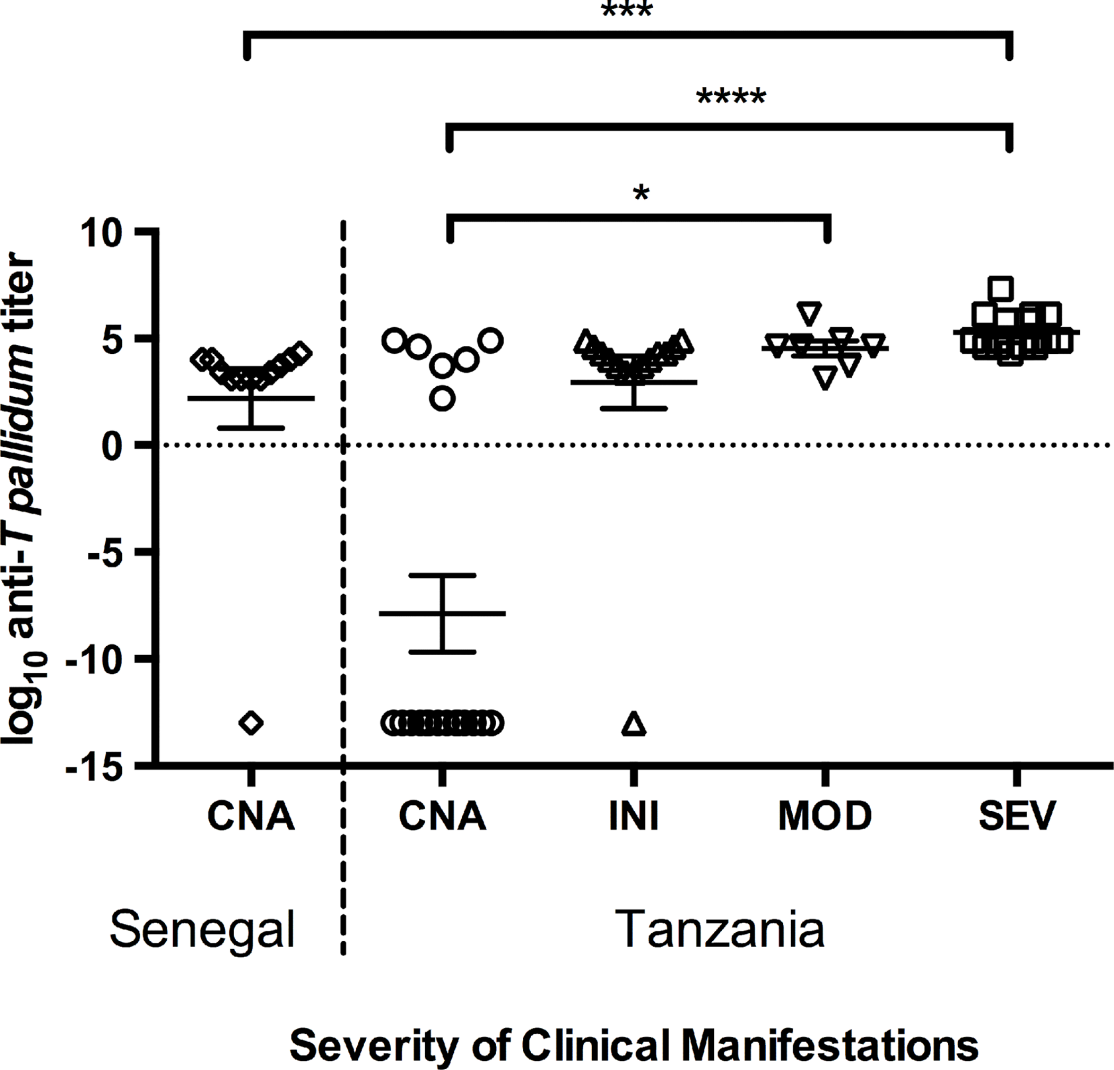
Overview of the anti-*T*. *pallidum* antibody titers of the Senegalese baboons sampled in 2013 compared to antibody titers obtained from *T*. *pallidum* infected baboons at Lake Manyara National Park in Tanzania [12]. Anti-*T*. *pallidum* antibody quantification was investigated using the Serodia TP-PA. Multiple comparison of log anti-*T*. *pallidum* titers in the group of baboons from PNNK in Senegal (CNA = clinically non-affected (n = 12), and four groups of baboons from Lake Manyara National Park in Tanzania with different stages of genital ulceration (CNA (n = 20), INI = initially (n = 14), MOD = moderately (n = 7), and SEV = severely genital-ulcerated (n = 16); data from [11], for stage definition see [12]). Kruskal-Wallis test using Dunn’s correction for multiple comparison: CNA (Tanzania) vs. SEV mean rank diff. = -38.96, ****p < 0.0001; CNA (Senegal) vs. SEV mean rank diff. = -31.23, ***p = 0.0004; CNA (Tanzania) vs. MOD mean rank diff. = -26.97, *p = 0.0198. (Mean ± SEM)

In all sero-positive baboons that were sampled in 2013, antibody titers had a median titer of 1:2,560 with a range of 1:1,280 to 20,480 (S1 Table). The log_10_-transformed data for the antibody titers obtained from the PNNK baboons did not follow a Gaussian distribution and thus were analyzed using a non-parametric test. The comparison of anti-*T*. *pallidum* antibody titers obtained from clinically non-affected (CNA) sero-positive Guinea baboons showed no significant difference to the results of CNA olive baboons at Lake Manyara National Park in Tanzania, as they are published elsewhere ([12], Fig 1). However, there is a tendency of higher antibody titers in the CNA baboons from Senegal with a median of 1:2,560 (range 0 to 1:20,480) compared to the titers of CNA Tanzanian baboons (median = 0, range 0 to 1:81,920). The same applies when compared to the group of initially (median = 1:15,360, range 0 to 1:81,920) and moderately genital-ulcerated and *T*. *pallidum* infected baboons of East Africa (median = 1:40,960, range 1:1,280 to 1.311e+6). In our analysis, there was a significantly lower antibody titer in the baboons sampled in 2013 at PNNK compared to severely infected (SEV) baboons at Lake Manyara National Park (p = 0.0004, SEV median = 1:81,920, range 1:20,480 to 2.097e+7; Fig 1).

## Discussion

### Transmission of *T*. *pallidum* in Senegalese baboons remains elusive

*T*. *pallidum* infection in baboons has been reported in many areas of tropical Africa [14]. Although the high number of *T*. *pallidum* sero-positive Guinea baboons at PNNK (90%, n = 18/20) was expected based on reports of the 1960s to 70s [1–4], it is currently not clear how *T*. *pallidum* is transmitted within a population of clinically non-affected baboons. In humans, yaws affects primarily young children and is generally transmitted through direct skin contact [15]. In primary yaws, lesions can be found commonly on the legs and ankles but can also be on arms and face. The primary lesion usually heals up within 3–6 months and is followed by secondary lesions that result from lymphatic and hematogenous spread (reviewed in [15]). These lesions are teeming with spirochetes and support the transmission of the pathogen by close contact with a susceptible new host. However, skin ulcers in baboons have not been reported from PNNK since behavioral research started in 2007. We have not yet detected treponemal DNA from blood collected in 2013 and 2014, though analysis is currently ongoing. The possibility to not only identify pathogen specific antibodies against *T*. *pallidum*, but also to study the genetic information of the simian strain(s) circulating in south east Senegal, would provide a better understanding of disease ecology and transmission pathways.

All simian T. pallidum strains analyzed thus far are genetically closely related to human yaws-causing strains [6, 7] and the Fribourg-Blanc simian strain must be considered as the most likely candidate to infect the baboons at PNNK. Historically, it was isolated from a baboon in West Africa and infection was not commonly associated with clinical manifestations [2]. It is possible that some baboons did sero-convert before macroscopic lesions became visible. This, however, is mostly unlikely since baboons are long-term monitored and all animals had no history of skin lesions before and after blood was sampled in 2013 and 2014. PNNK’s habituated baboons allow researchers to approach them very closely, sometimes closer than 2 meters of distance. If present, skin lesions would have been identified during some of the daily focal observations.

In humans, *T*. *pallidum* is recognized as an obligate pathogen, which has a very low infectious dose of ~50 inoculated microorganisms (at least for the syphilis causing ssp. *pallidum*, [16]). Congenital infection is a common feature in syphilis [17] but rarely reported in yaws [18]. It is not known whether simian strains can be transmitted from an infected mother via the placenta to an unborn child, but neither at the PNNK nor in the clinically affected East African baboon population has an increase in neonatal or sub-adult mortality rate been noted. Future studies at PNNK should include representative sampling of all age classes and long-term serological monitoring of individuals, starting at an early age and considering pedigrees. This could help to identify possible patterns of sero-conversion and may contribute to the identification and modeling of possible transmission routes in Guinea baboons.

**Fig 2:**
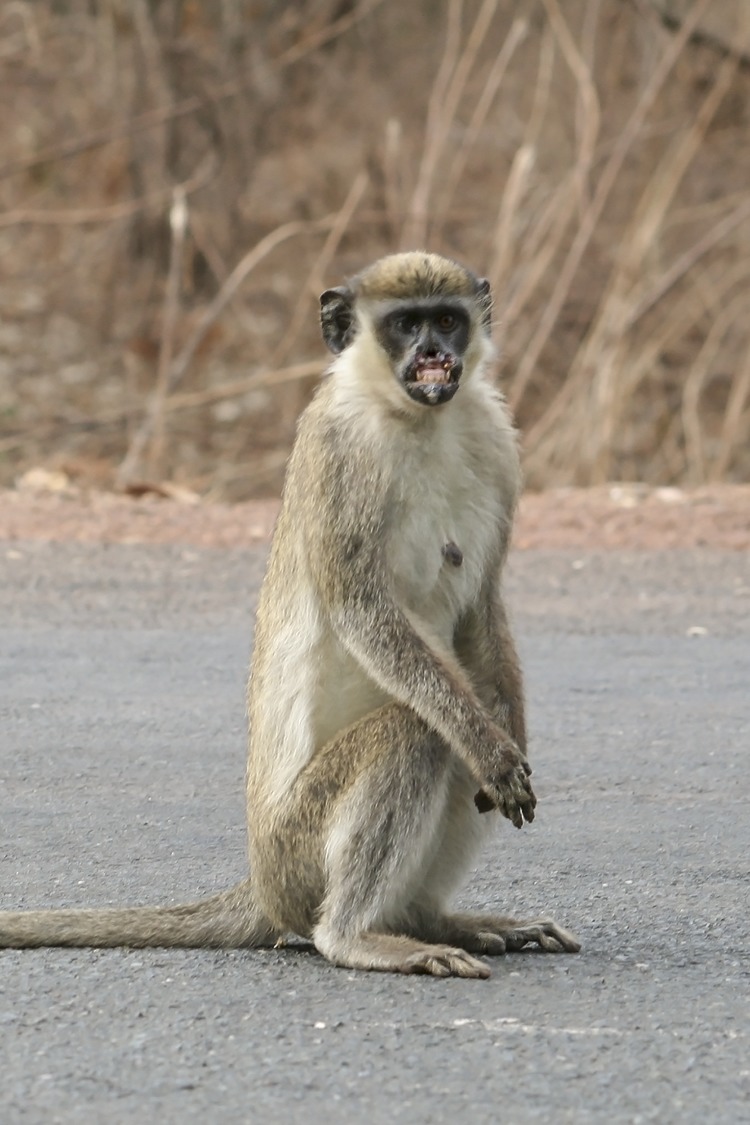
An adult green monkey (*Chlorocebus sabaeus*) with facial lesions at PNNK. The clinical manifestations resemble lesions known from tertiary human yaws infection.

Noteworthy, there is some evidence that other monkey species may in addition play a role as a reservoir for *T*. *pallidum* in Senegal. Interestingly, some green monkeys (*Chlorocebus sabaeus*) at PNNK do show signs of clinical infection that represent classic yaws-induce lesions in the face (Fig 2). A major task for future research activities must therefore include sampling of clinically infected green monkeys. Transmission between the latter and baboons may occur when juveniles play together or in the rare event, when adult baboons prey on green monkeys, as described for other baboon species [19]. It is possible that different species develop different clinical manifestations upon infection with the spirochete. This again underscores the importance to enhance research activities on transmission routs. Baboons and green monkeys tend to share resources (e.g., food and water) with humans. Even if monkey consumption has no recent history in Senegal, nonhuman primates may often be chased and killed to minimize crop raiding. At the same time, hunting provides a major potential source of cross-species infection with direct skin contact. The likelihood that infection in nonhuman primates is wide spread across West Africa, plus the tradition of primate bush meat hunting in neighboring countries such as Guinea, may provide a potential hotspot of nonhuman primate to human infection.

### Serology indicates a latent stage infection

All tests that were used in this study have been validated for the use in baboons [12]. Individual-based testing results of the TTs were constant for anti-*T*. *pallidum* antibodies of the IgG class in all assays. However, test results for IgM differed and were not always consistent. For example, one baboon was tested positive with the Mastafluor FTA-ABS IgM (case 6, Table 2, S1 Table), but tested negative with the Mastablot TP IgM. The opposite applied to case 11 (Table 2, S1 Table). All other animals were negative for IgM anti-*T*. *pallidum* antibodies. IgG antibodies against the spirochete are known to be stable and persistent throughout life [20], which correlates well with the reads of the IgG detecting TTs in this study. In contrast, IgM antibodies are the first to be produced post-infection but have a lower amplitude than IgG titers [21] and a reduced half-life compared to IgG [22]. In human treponematoses, antigen-specific IgM antibodies start to decline ~8 weeks post infection and may reach baseline levels after 2 years of infection [20]. Due to the high sero-prevalence of *T*. *pallidum* in baboons at PNNK, it can be assumed that infection is well established in the population and most animals are long-term infected with IgM titers that most likely reached baseline level already. However, it must be taken into consideration that at least some of the IgM results are false negative. IgM is sensitive to freeze-thaw cycles, which can contribute to the formation of cryoglobulins [23]. In this case, IgM may no longer be detected as it is precipitated. No free-floating detectable IgM will be found in such sera.

The inconsistent results of the cardiolipin EIA when compared to the corresponding NTT results (Table 2, S1 Table) may be due to the fact that cardiolipin is immobilized onto a solid phase using co-factors such as bovine sera. Co-factor related antigen structures, however, seem to be more specific for (human) phospholipid syndrome detection than for the use as a NTT. Thus, antibodies binding to VDRL and RPR may not be detected to the same level by an EIA.

Although Guinea baboons seem to develop no clinical symptoms of treponematosis, it is likely that infection is chronic or comparable to the latent stage in human yaws infection. This is supported by the low anti-*T*. *pallidum* serum titers in asymptomatic Guinea baboons (median = 1:2,560) *versus* serum titers that are found in genital-ulcerated baboons with active infection (median INI = 1:15,360, MOD = 1:40,960, and SEV = 1:81,920, [12]). Yet, low but consistent antibody levels again raise the question, why Senegalese Guinea baboons do not develop yaws- or syphilis-like lesions. This might be explained by strain specific genetic factors, which we do not understand yet and which are subject to functional analyses once the genomes of a substantial number of *T*. *pallidum* simian isolates have been sequenced and compared to human yaws and syphilis strains. In addition, host factors might be responsible for subtle changes in the immunological response, which is known to be a main driver for tissue damage during active infection in *T*. *pallidum* infection [24]. It is also possible that some species have coevolved with the bacterium and thus show different clinical manifestations through adaptation. However, Guinea baboons are parapatric with western olive baboons and hybridization among both species is likely [25–27]. It would thus be important to include western olive baboons in future investigations.

## Conclusion

Five decades after its first description from West Africa, south-eastern Senegalese Guinea baboons are (still) infected with *T*. *pallidum*, which is demonstrated by the specific antibodies against the spirochete. Though molecular detection of spirochete DNA is currently under investigation, the most likely strain that infects baboons in West Africa is the Fribourg-Blanc simian strain. This strain has been whole genome sequenced and shows close relationship to human yaws-causing *T*. *pallidum* strains [6]. In addition, it has been demonstrated to be infectious to humans [8]. This underlines the theoretic potential for Guinea baboons in West Africa, especially in areas where bush meat is consumed, to serve as a natural reservoir of human yaws infection. The reported results from our investigation in West Africa are of importance for the on-going second WHO led yaws eradication campaign [28] with its goal to eradicate yaws by 2020 [29].

## Supporting Information

S1 Table Individual-based dataset on clinical manifestations and serological test results. (XLSX)
